# Combined healthy lifestyle factors and psychosocial outcomes among cancer survivors: a systematic review and meta-analysis

**DOI:** 10.1007/s11764-024-01705-0

**Published:** 2024-11-09

**Authors:** Chunsu Zhu, Zhiwei Lian, Volker Arndt, Melissa S. Y. Thong

**Affiliations:** 1 Unit of Cancer Survivorship, German Cancer Research Center, Im Neuenheimer Feld 280, 69120 Heidelberg, Germany; 2 Medical Faculty, University of Heidelberg, Heidelberg, Germany

**Keywords:** Psychosocial outcomes, Lifestyle, Meta-analysis, Systematic review, Cancer survivor, Tertiary prevention

## Abstract

**Purpose:**

This systematic review aims to summarize the associations between combined healthy lifestyles and psychosocial outcomes (health-related quality of life (HRQOL), depression, anxiety, psychological distress (PD), and posttraumatic stress disorder (PTSD)) among cancer survivors.

**Methods:**

PubMed, Web of Science, Cochrane Library, and EMBASE were searched for observational and interventional studies examining healthy lifestyle scores (HLS, calculated by a combination of at least three lifestyles) and psychosocial outcomes among cancer survivors from inception to April 2024. A minimum of two studies with the same study design were pooled using random effects models.

**Results:**

Twenty-one studies (44,812 survivors) were included. Of all studies, 16 of which were included in meta-analysis. The pooling of cross-sectional evidence shows significant association between HLS and overall, physical, and psychosocial HRQOL. Significance was only observed for overall and physical HRQOL but not for psychosocial HRQOL in cohort studies. The estimations and 95% confidence interval (CI) with 1-point increase in HLS were 1.47 (0.83–2.12) and 1.42 (0.19–2.65) for overall and physical HRQOL, respectively. The evidence from interventional studies also indicated that interventions on multiple lifestyles have positive effects on the physical but not psychosocial HRQOL. Despite the limited number of studies, significant associations were found between HLS and depression, anxiety, PD, and PTSD.

**Conclusions:**

Although evidence is limited, we found that the combination of multiple healthier lifestyles is associated with better psychosocial outcomes in cancer survivors.

**Implications for cancer survivors:**

This review underscores the potential for adhering to multiple healthy lifestyles to improve psychosocial outcomes and enhance HRQOL for cancer survivors.

**Supplementary Information:**

The online version contains supplementary material available at 10.1007/s11764-024-01705-0.

## Introduction

Due to the increasing cancer incidence, population aging, and advancements in early screening and treatment, there is an increasing number of people living with a diagnosis of cancer [1]. According to recent estimates, there were approximately 20 million new cancer cases worldwide in 2022 [2]. An estimated of 18.1 million citizens in the United States were living with a cancer diagnosis in 2022 [3], and the number for Europe was close to 24 million in 2020 [4]. Cancer survivors are more likely to suffer from psychosocial problems (e.g., lower health-related quality of life [HRQOL], depression, anxiety, and psychological distress [PD] [5–7]) than the general population, even years after diagnosis. Improving survival has always been regarded as the key aim of cancer survivor care. However, another important but under acknowledged aspect is the psychosocial health care of cancer survivors. Psychosocial health is not only associated with HRQOL, but may also have an impact on the survival of cancer survivors [8].

Previous evidence has linked healthy lifestyles with the promotion of better psychosocial outcomes of cancer survivors [9, 10]. However, the majority of studies have focused on only one or two specific lifestyles, such as physical activity [11], diet [12], and body mass index (BMI) [13] and psychosocial outcomes. Considering that lifestyle factors often occur concurrently and interact with each other, it is uncommon for individuals to change an isolated health behavior in real life. Assessing multiple healthy lifestyle factors jointly may provide a more profound and precise understanding of the association between lifestyles and psychosocial outcomes among cancer survivors. There are a growing number of studies examining the relationship between combined healthy lifestyle factors and psychosocial outcomes among cancer survivors. However, the results are mixed [14, 15]. To the best of our knowledge, no systematic review and meta-analysis on this topic are available. To fill this gap, we conducted this systematic review and meta-analysis to summarize the evidence from observational and interventional studies on the relationship between combined healthy lifestyles and psychosocial outcomes among cancer survivors.

## Methods

This systematic review and meta-analysis were performed following the Preferred Reporting Items for Systematic Reviews and Meta-Analyses (PRISMA) [16] checklist and is registered at the International Prospective Register of Systematic Reviews (CRD42024544379).

### Search strategy

PubMed, Web of Science, Cochrane Library, and EMBASE were searched from inception to April 2024 by CZ with search terms related to “combined,” “lifestyle,” “cancer survivor,” “depress*,” “anxiety,” “quality of life,” “mental health,” “well-being,” and “psychosocial outcomes” included in the titles or abstracts. The detailed search strategies are provided in supplementary Table S1. The reference lists of relevant reviews and included studies were checked manually to identify additional studies.

### Study selection

After removing duplicate studies, CZ and ZL carried out the study selection independently according to the following inclusion and exclusion criteria. Any controversies were addressed by consulting a third investigator (MT).

The inclusion criteria were as follows: (1) were randomized controlled trials (RCTs) or observational studies (cohort or cross-sectional studies) investigating the association between combined healthy lifestyle factors and psychosocial outcomes among cancer survivors; (2) used a combination of at least three healthy lifestyle factors as interventions or exposures, and the lifestyle factor included but not limited to physical activity, diet, cigarette smoking, alcohol consumption, and body weight; (3) the primary psychosocial outcomes of interest was HRQOL, the secondary psychosocial outcomes of interest were depression, anxiety, post-traumatic stress disorder (PTSD), and PD; and (4) focused on individuals with a diagnosis of cancer, regardless of the cancer sites and stages; (5) age at diagnosis ≥ 18 years; (6) language was English.

The exclusion criteria were as follows: (1) reviews, abstracts, case studies, and animal studies; (2) publications that used a combination of only one or two healthy lifestyle factors as the interventions or exposures because we assumed that the overall lifestyle patterns could not be fully reflected by only one or two lifestyle factors; (3) studies that focused on the general population or individuals with a diagnosis of diseases other than cancer; and (4) duplicate studies.

### Data extraction and study quality assessment

The data were extracted by CZ and ZL independently using a predesigned extraction form. The following data were extracted: name of the first author, publication year, title, country, characteristics of the study population (cancer type, age, sex), sample size, follow-up duration (only for longitudinal studies), definition and measurements of outcomes, components and definition of combined healthy lifestyle factors, selection or intervention of control groups, adjusted confounding factors, and adjusted risk estimates with corresponding 95% confidence intervals (CIs).

All included studies in this systematic review and meta-analysis were evaluated using standardized and well-used tools. The National Institute of Health Quality Assessment Tool for Observational Cohort and Cross-sectional Studies (NIH) was utilized to assess the quality of cross-sectional studies and cohort studies [17], and the Cochrane Risk-of-Bias Assessment Tool was used for interventional studies [18]. The NIH contains 14 items and evaluates the study quality through the assessment of the possible sources of bias (e.g., selection, information, measurement), confounding factors, reporting, and other factors (supplementary Table S2). Among the 14 items, only 8 are relevant to cross-sectional studies [19]. Hence, only 8 out of 14 items were applied for cross-sectional studies in our study. For each item, a positive response was assigned 1 point, while 0 points were given to negative or uncertain answers. A total quality score was created for the included studies by summing scores across each item, with a higher score indicating better study quality [19]. For cohort studies, a score ≥ 11 out of 14 indicated excellent quality; for cross-sectional studies, a score ≥ 6 out of 8 points indicated good study quality [20].

### Statistical analysis

#### *Meta*-analysis

All meta-analyses were carried out in R software using the metafor package, and a two-sided *p*-value < 0.05 was regarded as statistically significant. The summarized effect size and 95% CI were calculated for studies with the same study design and same outcomes, using random effects models, allowing heterogeneity across studies. There are three types of healthy lifestyle score (HLS), including the basic summing score (assuming that each factor has an equal impact on psychosocial outcomes, and a certain point was assigned to participants with or without a healthy lifestyle) [21]; the World Cancer Research Fund and the American Institute for Cancer Research (WCRF/AICR) score which comprises body fatness, physical activity, energy-dense foods, plant foods, meat consumption alcoholic drinks, and dietary supplements [22]; and the American Cancer Society (ACS) score which includes BMI, physical activity, and the intake of vegetables and fruits, proportion of total grains, and red and processed meat [23]. To reduce the heterogeneity and improve the comparability across studies, we reported the results of meta-analyses by different types of HLS. Mean differences (MDs) and 95% CIs were estimated to show the pooled intervention effects for interventional studies. The pooled beta coefficient, depicting the average increase in outcome scores with per-unit increase in the HLS, was calculated for observational studies. When studies did not report linear regression results, we calculated the beta coefficient by the weighted linear regression method using the number of survivors in each group or the inverse variance as weights, where possible, following previous studies [19]. If results were provided for patterns of HLS, such as 0–2 and 5–6, the midpoints were adopted. The HRQOL score was aggregated in three levels. Studies that provided an overall HRQOL score were pooled in a global HRQOL meta-analysis. For studies without a summary score, the results were synthesized on a physical and a psychosocial component score level, following the dimensions of 36-item versions of the Medical Outcome Study Short-Form [24]. For studies that only provided scale scores for adopted HRQOL tools (e.g., physical functioning, role functioning, social functioning), multiple dependent effect sizes, which could reduce the bias in the combined effect size, were summarized using the methods proposed by previous studies [25]. The intercorrelations for HRQOL tools were taken from published studies [24, 26]. For the other psychosocial outcomes of interest, a meta-analysis was carried out only when there are at least two studies reporting the same outcomes with the same tools and the same type of HLS. Small study bias (e.g., publication bias) was examined by Egger’s test and visualization of funnel plots when at least 10 studies were pooled. Heterogeneity was assessed by *I*^2 statistics (ranging from 0 to 100%) and *P* values for the Cochran *Q* test. According to guidelines from the Cochrane Handbook for Systematic Review, *I*^2 values ranging from 0 to 40%, 30 to 60%, 50 to 90%, and 75 to 100% were considered to reveal low, moderate, substantial, and considerable heterogeneity, respectively[27].

### Summary methods for studies not included in the *meta*-analysis

For studies that met our inclusion criteria but were not included in the meta-analysis due to differences in study design and outcome measurement tools, we provide a brief summary of the findings pertinent to our research question.

## Results

### Study selection

**Fig. 1:**
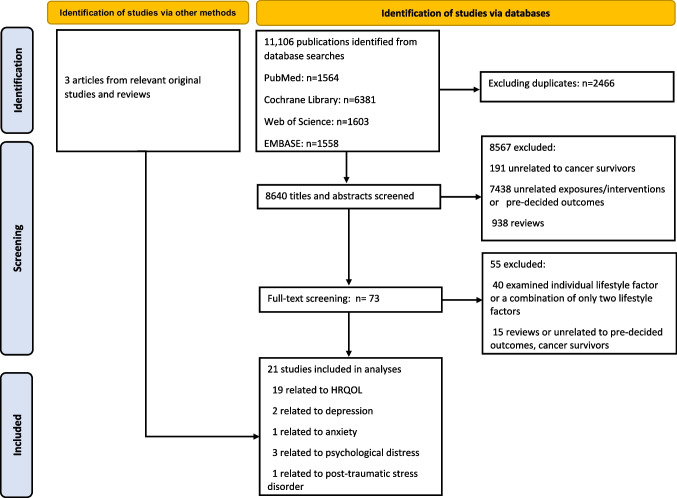
The flowchart of study selection. HRQOL, health-related quality of life

The study selection process is presented in Fig. 1. Briefly, a total of 11,106 items were identified from PubMed, Cochrane Library, Web of Science, and EMBASE. After removing the duplicates, 8640 titles and abstracts were screened. The majority of studies were excluded because they were reviews, unrelated to the exposures/interventions or outcomes of interest, or were focused on non-cancer individuals, leaving 73 studies for full-text reading. We further excluded 55 studies that either investigated a combination of only one or two lifestyles as the exposure or intervention, or unrelated to cancer survivors. Additionally, three studies were identified by scanning the references of the included studies and related reviews. Finally, 21 studies were selected for this systematic review and meta-analysis.

### Characteristics of included studies

In total, 21 quantitative studies, covering 44,812 cancer survivors, were included (Table 1). Most studies were published within the last decade (*n* = 16). Fourteen were cross-sectional studies [15, 28–40], five were cohort studies [41–45], and two were RCTs [46, 47]. All studies were conducted in high-income regions (the USA = 8 [15, 28, 29, 32, 34, 37, 38, 40], Netherlands = 4 [31, 33, 41, 43], Germany = 2 [36, 44], the UK = 1 [39], Australia = 2 [46, 47], Canada = 1 [30], South Korea = 2 [35, 42], China Hong Kong = 1 [45]). Of the studies, seven were conducted in colorectal cancer survivors [31, 33, 36, 39, 43, 44, 47], six were in mixed cancer survivors [28, 29, 32, 37, 40, 46], two were in breast cancer survivors [35, 45], two were in bladder cancer survivors [30, 41], and one study was in survivors diagnosed with other cancer types (including gastric cancer, cervical cancer, endometrial cancer, and non-Hodgkin lymphoma) [15, 34, 38, 42]. Ten studies were among survivors diagnosed within 5 years since diagnosis, 10 studies were on long-term cancer survivors (at least 5 years postdiagnosis), and the duration since cancer diagnosis was unclear in one study. The sample size ranged from 120 to 18,902. The mean age of cancer survivors at enrollment varied between 32.4 and 78.9 years, and the proportion of male survivors ranged from 0 to 80.0%.

**Table 1:** Characteristics of included studies

Author (year)	Study design	Country	Outcomes	Outcome scale	Cancer type	Sample size	Mean age, *y*	Male (%)	Maximum follow-up duration	Average duration post diagnosis, *y*
Vidra (2023) [41]	Cohort	Netherlands	HRQOL	EORTC QLQ-C30	NMIBC	1029	65.9	826 (80)	Up to 1.25 years	Up to 1.25
Kim (2023) [42]	Cohort	South Korea	Depression	ICD‐10	GC	18,902	40 +	13,705 (72.5)	Up to 4.7 years	Unclear
Kenkhuis (2022) [43]	Cohort	Netherlands	HRQOL	EORTC QLQ-C30	CRC	396	67.0	270 (68.2)	Up to 2 years	Up to 2
Eyl-Armbruster (2022) [44]	Cohort	Germany	HRQOL	EORTC QLQ-C30	CRC	2283	66.0	1415 (62)	10 years	≥ 5.0
Lei (2018) [45]	Cohort	China	HRQOL	EORTC QLQ-C30	BC	1462	53.7	0	Up to 1.5 years	Up to 1.5
Hawkes (2013) [47]	RCT	Australia	HRQOL	SF–36	CRC	410	66.4	221 (53.9)	1 year	0.5
Seib (2022) [46]	RCT	Australia	HRQOL	SF-36	Mixed	351	53.2	0	24 weeks	1.58
Olson (2023) [28]	Cross-sectional	USA	HRQOL	SF-12	Mixed	591	66.0	215 (36.94)	NA	1.5
Glasgow (2022) [29]	Cross-sectional	USA	PD	PHQ-4	Mixed	856	63.3	376 (43.75)	NA	12.29
Zhang (2018) [32]	Cross-sectional	USA	HRQOL	SF-36	Mixed	2480	32.4	1,275 (51.4)	NA	24.1
Breedveld-Peters (2018) [33]	Cross-sectional	Netherlands	HRQOL PD	EORTC QLQ-C30 HADS	CRC	155	70.0	91 (63)	NA	5.7
Iyer (2016) [15]	Cross-sectional	USA	QOL Depression Anxiety PD	FACT-Cx PROMIS BSI	CC	204	43.2	0	NA	1.5
Spector (2015) [34]	Cross-sectional	USA	HRQOL PTSD	SF-36 PCL-C	NHL	566	67.2	272 (48.1)	NA	15.2
Schlesinger (2014) [36]	Cross-sectional	Germany	HRQOL	EORTC QLQ-C30	CRC	1389	67.0	702 (50.1)	NA	6.0
Inoue-Choi (2013) [37]	Cross-sectional	USA	HRQOL	SF–36	Mixed	2193	78.9	0	NA	8.9
Gruenigen (2011) [38]	Cross-sectional	USA	QOL	SF–36	EC	120	56.8	0	NA	< 5.0
Grimmett (2011) [39]	Cross-sectional	UK	QOL	EORTC QLQ-C30	CRC	478	67.8	284 (59.4)	NA	< 5.0
Blanchard (2008) [40]	Cross-sectional	USA	HRQOL	RAND-36	Mixed	9105	67.4	3147 (34.6)	NA	Up to 10
Veen (2019) [31]	Cross-sectional	Netherlands	HRQOL	EORTC QLQ-C30	CRC	1096	70.8	635 (58)	NA	8.1
Song (2015) [35]	Cross-sectional	South Korea	HRQOL	EORTC-QLQ-30	BC	160	51.0	0	NA	Unclear
Chung (2020) [30]	Cross-sectional	Canada	HRQOL	BUSS	MIBC NMIBC	586	67.3	401 (68.4)	NA	3.47

SF-36, 36-item version of the Medical Outcome Study Short-Form; SF-12, 12-item version of the Medical Outcome Study Short-Form; PHQ-4, Patient Health Questionnaire-4; EORTC QLQ-C30, European Organization for the Research and Treatment of Cancer Quality of Life Questionnaire-Core 30; HADS, Hospital Anxiety Depression Scale; PROMIS, Patient‐Reported Outcomes Measures Information System; BUSS, 10-item,validated Bladder Utility Symptom Scale; ICD-10, International Classification of Diseases, 10th revision; FACT-Cx, Functional Assessment of Cancer Therapy-Cervical questionnaire; PCL-C, Posttraumatic Stress Disorder Checklist-Civilian form; RAND-36, Health Status Inventory; HRQOL, health-related quality of life; PTSD, posttraumatic stress disorder; GC, gastric cancer; CRC, colorectal cancer; BC, breast cancer; EC, endometrial cancer; CC, cervical cancer; NHL, non-Hodgkin lymphoma; NMIBC, non-muscle invasive bladder cancer; MIBC, muscle-invasive bladder cancer; BSI, Brief Symptom Inventory; PD, psychological distress

### Measurements of outcomes

HRQOL was measured by the European Organization for the Research, the Treatment of Cancer Quality of Life Questionnaire-Core 30 (EORTC QLQ-C30, *n* = 9) [31, 33, 35, 36, 39, 41, 43–45], the 12-item or the 36-item versions of SF (SF-36, *n* = 6 [32, 34, 37, 38, 46, 47]; SF-12, *n* = 1 [28]), the 10-item Bladder Utility Symptom Scale (BUSS, *n* = 1) [30], the Functional Assessment of Cancer Therapy-Cervical questionnaire (FACT-Cx, *n* = 1) [15], and the RAND-36 Health Status Inventory (RAND-36, *n* = 1) [40]. Depression was ascertained by the Patient‐Reported Outcomes Measures Information System (PROMIS, *n* = 1) [15] and the International Classification of Diseases, 10th revision (ICD-10, *n* = 1) [42]. Anxiety was assessed using the PROMIS (*n* = 1) [15], PD was defined by the Brief Symptom Inventory (BSI, *n* = 1) [15], the Patient Health Questionnaire-4 (PHQ-4, *n* = 1) [29], and the Hospital Anxiety Depression Scale (HADS) [33], and PTSD was assessed with the Post-traumatic Stress Disorder (PTSD) Checklist-Civilian form (PCL-C, *n* = 1) [34].

### Assessment of combined healthy lifestyles

The most common factors in the combined HLS were physical activity, diet, body weight, alcohol consumption, and cigarette smoking (Supplementary Table S3). Three studies also included sleep and sedentary behavior. Seven studies reported the reported WCRF/AICR score [31, 33, 35, 37, 41, 43, 45], nine studies reported the basic summing score [15, 29, 30, 36, 38–40, 42, 44], and three studies reported the ACS score [32, 34, 35].

### Quality of the included studies

The results of the quality assessment are provided in supplementary Tables S4-S5. Four cohort studies were of excellent quality. Fourteen cross-sectional studies were of good study quality. The main reasons for lower quality scores were the following: (1) a participation rate of at least 50%; (2) no sample size justification; (3) no clear definition of exposures; and (4) non-adjustment for potential confounding factors.

The quality of the interventional studies was assessed using the Cochrane Risk-of-Bias Assessment Tool, and the results were presented in supplementary Table S6. Overall, one RCT was assessed as “having some concerns” because the intervention group was younger than the control group which may lead to an overestimation of the interventional effects [47], and one was regarded as having “high-risk bias” because there is no mention of allocation concealment and the intervention was not blind to the participants [46].

### Combined healthy lifestyles and quality of life among *cancer* survivors

**Fig. 2:**
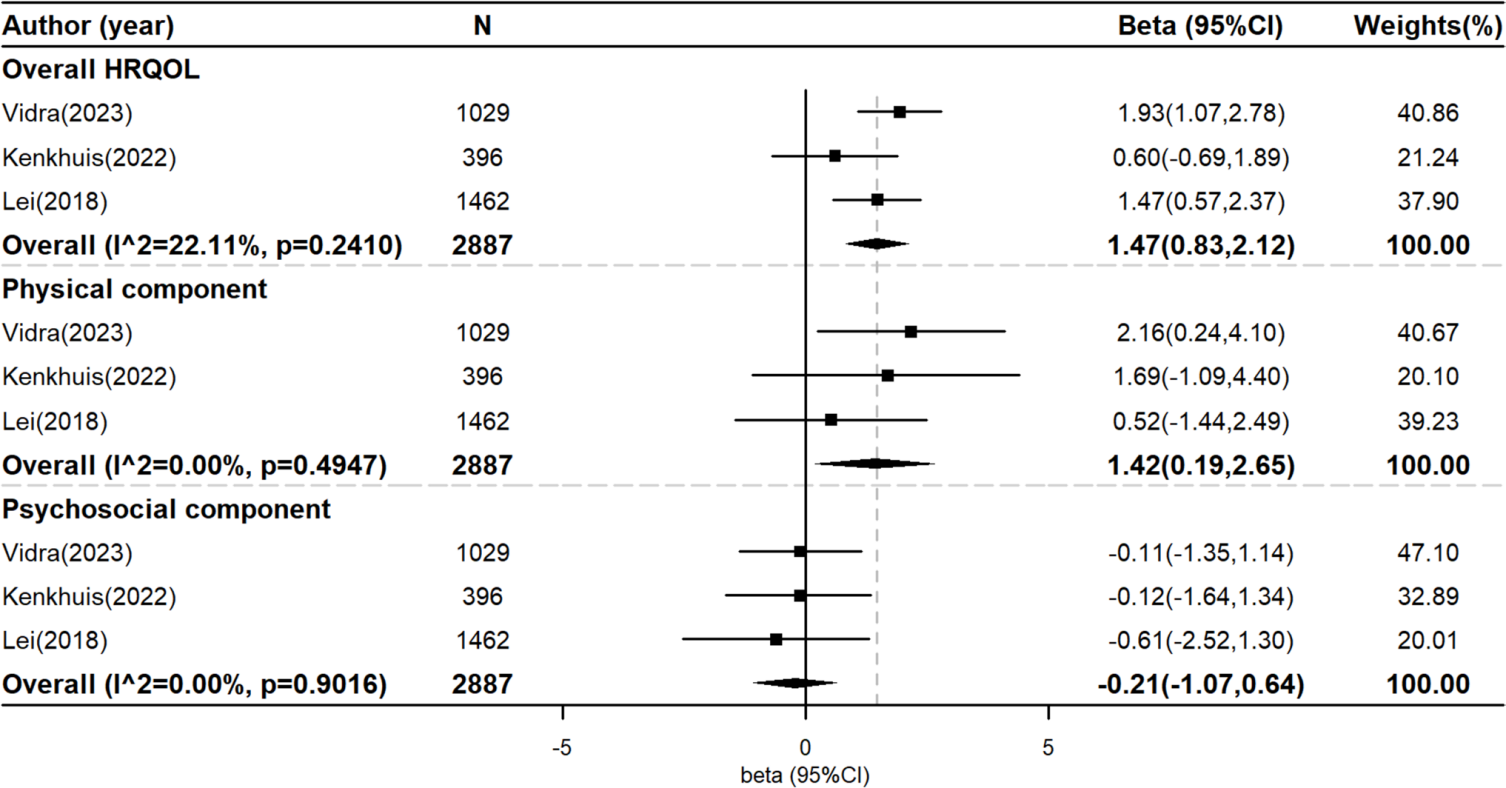
Meta-analysis on cohort studies examining the association between healthy lifestyle score and quality of life. This figure presents the pooled beta coefficient from cohort studies assessing the relationship between a healthy lifestyle score and HRQOL. Each beta coefficient represents the average increase in HRQOL scores with per-unit increase in the healthy lifestyle score, with 95% confidence intervals (CIs) depicted for each study. Heterogeneity across studies was assessed using *I*^2 statistics and *P* values for the Cochran *Q* test. A positive beta coefficient indicates an improvement in HRQOL with higher healthy lifestyle scores. HRQOL, health-related quality of life; CI, confidence interval

**Fig. 3:**
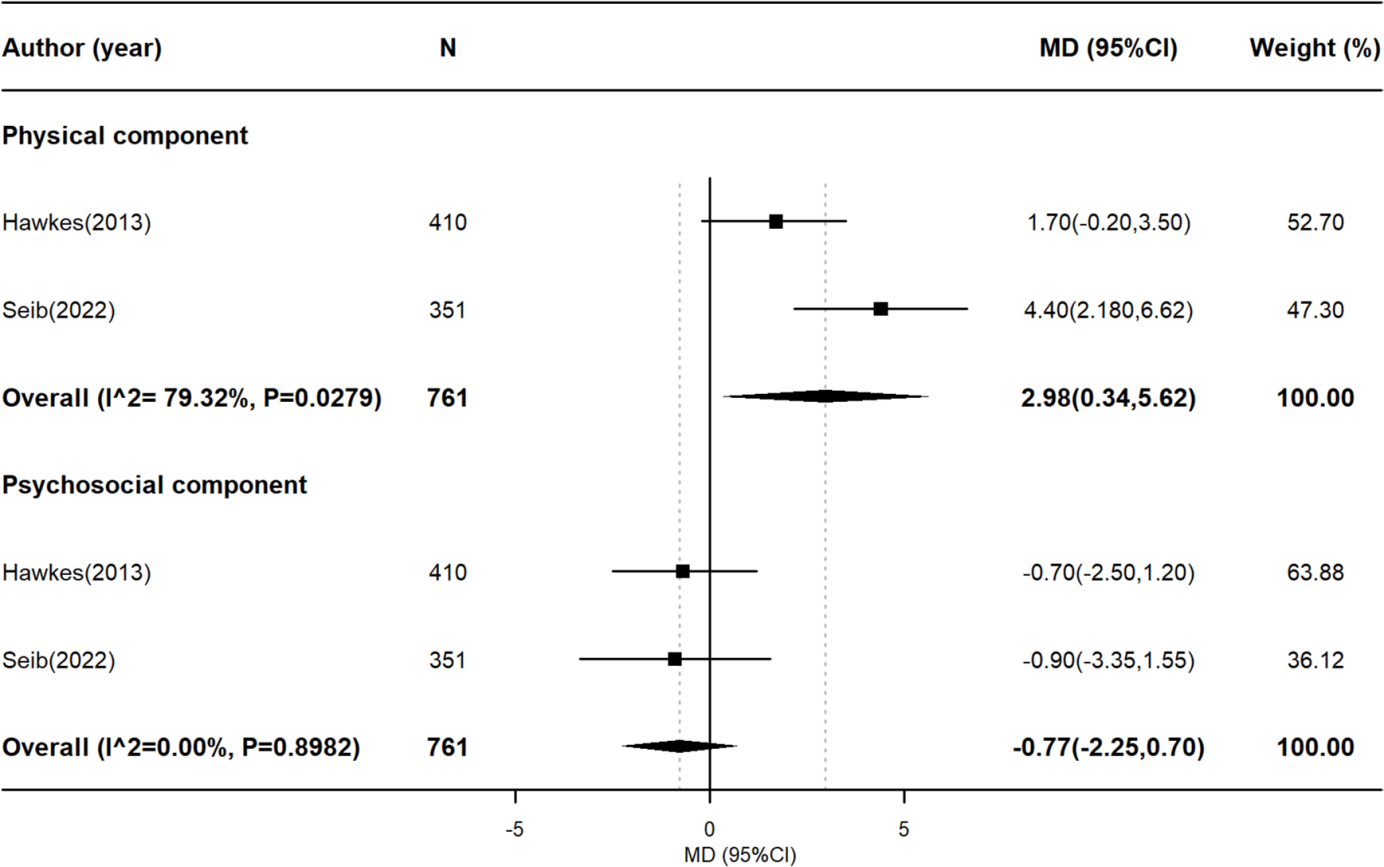
Meta-analysis on interventional studies examining the association between healthy lifestyle score and quality of life. This figure presents the pooled effect size (mean difference, MD) from interventional studies examining the impact of multiple lifestyle behavior interventions on HRQOL. A positive MD indicates that survivors in the intervention group experienced better HRQOL compared to the control group. Heterogeneity across studies was assessed using *I*^2 statistics and *P* values for the Cochran *Q* test. HRQOL, health-related quality of life; MD, mean difference; CI, confidence interval

Of 19 studies that examined the association between combined healthy lifestyle factors and HRQOL, 16 studies (2 RCTs, 3 cohorts, and 11 cross-sectional studies) were included in the meta-analysis. The evidence from the cross-sectional studies is summarized in supplementary Figure S1, and it was shown that there are significant associations between WCRF/AICR score and overall (beta = 1.13, 95% CI = 0.49–1.77), physical (beta = 1.86, 95% CI = 1.63–2.09, *n*), and psychosocial HRQOL (beta = 0.65, 95% CI = 0.13–1.18). However, the meta-analyses of cohort studies [41, 43, 45] (2887 survivors) only found a significant relationship between WCRF/AICR score and overall HRQOL and physical HRQOL, but not psychosocial HRQOL. All HLS in cohort studies were calculated following WCRF/AICR recommendations. Each one-unit increase in WCRF/AICR score was related to 1.47 and 1.42 increase in overall HRQOL (beta = 1.47, 95% CI = 0.83–2.12) and physical HRQOL (beta = 1.42, 95% CI = 0.19–2.65), respectively (Fig. 2). Although only a small number of participants were included in the RCTs (*n* = 761 survivors) [46, 47], the synthesized results also found similar results, with an improvement in physical HRQOL among cancer survivors and no significant effect for psychosocial HRQOL (Fig. 3).

Three studies (1 cohort and 2 cross-sectional studies) were not included in the meta-analysis due to the missing of a necessary parameter. Of these studies, the cross-sectional studies showed that survivors who were engaged in a higher number of healthy lifestyles reported better HRQOL [28, 36]. A cohort study of 2283 long-term colorectal cancer survivors examined the associations between the baseline score and changes in the HLS and HRQOL. It found that a low HLS at baseline was associated with poorer functioning, lower global HRQOL, and more symptoms in comparison with high baseline HLS, and survivors who improved their overall HLS from baseline to 5-year follow-up reported better functioning and fewer symptoms [44]. In terms of the relationship between changes in the HLS and HRQOL, another study also reported that improvement in the adherence to WCRF/AICR recommendations from baseline to 18-month follow-up after cancer diagnosis was associated with higher HRQOL scores [45]. However, the results from two cohort studies suggested that these associations were more likely to be driven by between-subject differences [41, 43]. A prospective cohort study of 459 colorectal cancer survivors collected lifestyle data (including dietary intake, body composition, sedentary behavior, and physical activity) at 6 weeks, 6, 12, and 24 months posttreatment [43]. In that study, a higher HLS was associated with better physical functioning and less fatigue in the inter-individual, but not the intra-individual analyses [43]. Similar results were observed among non-muscle invasive bladder cancer survivors in the first 15 months after diagnosis [41].

### Combined healthy lifestyles and other psychosocial outcomes among *cancer* survivors

The number of studies that reported on other psychosocial outcomes (including depression, anxiety, PD, and PTSD) was not sufficient to perform a meta-analysis. Therefore, we summarized their findings. Two studies (1 cohort and 1 cross-sectional study) examined the associations between a combination of at least three healthy lifestyle factors and depression among cancer survivors. Using the Korean National Health Insurance Service databases, a study examined the effect of multiple healthy lifestyles (including smoking cessation, alcohol abstinence, and starting regular physical activity) on new-onset depression among 18,902 gastric cancer patients and found that the risk of depression tended to decline as HLS increased [42]. Compared to the reference group (0 point), the HRs and 95% CI for 1 point, 2 points, and 3 points groups were 0.69 (0.55–0.83), 0.60 (0.50–0.76), and 0.55 (0.45–0.68), respectively [42]. In addition, gastric survivors who previously had all three unhealthy lifestyles but changed to healthier lifestyles experienced a reduced depression risk of 50% [42]. A cross-sectional study among 204 cervical cancer survivors also reported a significant association between the number of healthy lifestyle factors and depression or anxiety scores [15]. Three cross-sectional studies explored the association between the combination of at least three lifestyle factors and PD in cancer survivors. A cross-sectional study of 856 cancer survivors reported that survivors who met the recommendations for seven health behaviors experienced improved psychosocial health and survivors who met more health behaviors reported lower PD [29]. Two studies reported that a higher PD score was observed among those who had more unhealthy lifestyle factors [15, 33]. A cross-sectional study conducted on 566 individuals living with a history of non-Hodgkin lymphoma found that survivors who met the ACS guidelines regarding physical activity, fruit and vegetable consumption, body weight, and tobacco use reported lower PTSD [34]. The beta coefficient for survivors who met 4, 3, and 2 healthy lifestyle guidelines was − 0.41. − 0.30, and − 0.28, respectively, compared to those who met none of the guidelines [34].

## Discussion

In this systematic review and meta-analysis, we summarized the findings from 21 publications that investigated the adoption of multiple healthy lifestyles and psychosocial outcomes in cancer survivors. Overall, the results suggest that adherence to multiple healthy lifestyle factors is significantly associated with better psychosocial outcomes in cross-sectional, cohort, and interventional studies.

To the best of our knowledge, our review is the first to summarize the evidence on associations between a combination of at least three lifestyle factors and HRQOL in cancer survivors. In general, we found that adherence to multiple healthy lifestyles was associated with better HRQOL in both observational and interventional studies. Although no review has examined the relationship between HLS and HRQOL in cancer survivors, the relationships of individual lifestyle factors, such as exercise [48], diet [49], and body weight [13], with HRQOL are well understood. Using a network meta-analysis method, a recent review compared the effects of lifestyle/behavioral interventions on HRQOL in breast cancer survivors and reported that aerobic-resistance exercise might be the most effective intervention for improving HRQOL [50]. The effect of physical activity/exercise on HRQOL in cancer survivors has been reported in several reviews [51, 52]. In addition, a meta-analysis of nine experimental studies demonstrated that dietary intervention could significantly improve the overall HRQOL score [49]. Reviews on the relationships between a combination of two lifestyle factors (mainly physical activity/exercise and diet) and HRQOL in cancer survivors reported mixed results. A meta-analysis of two RCTs in gynecological cancer survivors showed that combined diet, exercise, and cognitive behavioral interventions had no significant impact on HRQOL [53]. In contrast, a review on exercise/nutrition programs and HRQOL among older adults living with and beyond cancer demonstrated that half of the included studies indicated a positive association [54]. A recent systematic review of nine RCTs concluded that a combination of physical activity/exercise and diet/nutrition may improve the physical but not the mental component of HRQOL in cancer survivors [55], which is in line with our results to some extent. In our review, the evidence from cohort studies and RCTs suggests that combined healthy lifestyles had no significant effect on the psychosocial aspect of HRQOL in cancer survivors. The reasons for this nonsignificant association between multiple healthier lifestyles and psychosocial QOL are unclear but might involve the following explanations. First, it is possible that the mental stress associated with a cancer diagnosis and its treatment may have reduced in the interim period before the start of the investigation. For example, the survivors in the two RCTs were recruited 12 or 19 months after diagnosis [46, 47]. In one RCT, only survivors who had completed the cancer treatment were included, and the mental health summary score improved not only in the intervention group but also in the control group [46]. Second, the participants included in the studies were relatively healthier and tended to have better baseline HRQOL and higher awareness towards healthy lifestyles. For instance, in one RCT of colorectal cancer survivors, only those who had no metastatic disease and no medical conditions that could affect the adherence to a healthy lifestyle were included. The ceiling effects may restrict the ability of the authors to detect the differences. Future interventional investigations may consider recruiting survivors who have unhealthier lifestyles and poor HRQOL. Third, some lifestyle factors, such as physical activity, are more important than other healthy lifestyles in affecting HRQOL [50, 56]. However, the majority of studies assumed that all healthy lifestyles have the same impact on HRQOL. This may underestimate the effect of these important factors. In addition, the interventions may not achieve the expected effects on the improvement of these healthy lifestyles. In one RCT, limited improvements in physical activity were observed in the intervention group [47]. The lack of interventional effects on healthy lifestyles may also contribute to the nonsignificant associations with mental HRQOL.

We also explored the associations of healthier lifestyles with other psychosocial outcomes including depression, anxiety, PD, and PTSD, but the number of studies is limited. Results from a cohort study and a cross-sectional study revealed that depression decreased in parallel with the increase of healthy lifestyles among cancer survivors [15, 42]. Despite the limited evidence, we speculate that cancer survivors could benefit from adhering to multiple healthy lifestyles to reduce depressive symptoms. Previous evidence supports our speculation. For instance, reviews have shown that a combination of physical activity and diet intervention have protective effects on depression among cancer survivors [55]. In addition, a meta-analysis among the general population reported that a combination of at least three healthy lifestyles was associated with reduced depressive symptoms, suggesting the importance of adopting multiple healthy lifestyles in the prevention of depression [57]. Our review also revealed a significant relationship between combined healthy lifestyle factors and anxiety, PD, and PTSD. However, as the results were from only one or two low-quality cross-sectional studies, further validation of the results using more robust designed studies is needed.

This review comprehensively summarized the evidence on the role of combined healthy lifestyle factors in psychosocial health among cancer survivors. It provided meaningful insights into the influence of adherence to multiple healthy lifestyles on psychosocial outcomes for cancer survivorship. By using systematic review and meta-analysis methods, this review could also identify the directions for future research. For example, very few longitudinal studies were identified in the current review, and the outcomes measurement scales used varied across studies. More longitudinal studies and intervention trials, with validated study designs and consistent measurement tools for psychosocial outcomes among cancer survivors, are warranted.

However, this review has several limitations. The main limitation is the limited number of studies with appropriate data for certain psychosocial outcomes (e.g., depression and anxiety) among cancer survivors, restricting our ability to perform a meta-analysis of these outcomes. Additionally, due to the differences in study designs and outcome scales, and the missing of necessary parameters, not all included publications could be meta-analyzed. Nevertheless, we provided a summary narrative that distilled important insights into these associations. Second, most of the included studies were cross-sectional, and the number of high-quality cohort studies and RCTs was small. This could limit the causal inference in our review because healthy lifestyle behaviors and psychosocial outcomes, such as HRQOL, might mutually affect one another [58]. Third, there was heterogeneity across studies for some results, which could be explained by the different characteristics of the study populations (e.g., age, cancer types, and cancer stages), diverse study methodology (e.g., outcome measurement tools, follow-up periods, and adjusted confounding factors), the various components of lifestyle factors, and different scoring methods used to calculate the HLS. Two main types of HLS, the basic summing score and the WCRF/AICR score, were summarized in the current review. Both are unweighted, assuming that each lifestyle factor contributes equally and additively to the outcomes. Despite this, these scores have notable strengths. The basic summing score is straightforward to calculate, making it easy to communicate with patients in clinical practices. The WCRF/AICR score is developed through a comprehensive literature review of available data. It includes two versions: the 2007 and 2018 WCRF/AICR scores [59, 60], with the latter one being an update of the earlier one. The 2018 WCRF/AICR provides a standard scoring system, created through the collaboration of the US National Cancer Institute, the members of WCRF/AICR, and other international researchers. Although the WCRF/AICR score does not address certain major cancer risk factors, such as smoking and abdominal adiposity, future investigations using this new scoring system may lead to more robust findings. Fourth, the included studies were mainly from high-income countries, and the results should be interpreted with caution when generalizing to other countries. Finally, despite our efforts to find unpublished results, the number of studies involved in all pooled analyses was less than ten; thus, the estimation of publication bias was not conducted.

## Conclusions

In conclusion, this systematic review and meta-analysis indicated that adherence to multiple healthy lifestyles is associated with better psychosocial outcomes among cancer survivors. Although the number of included studies was small, the findings highlight the potential benefits of adhering to an overall healthy lifestyle for better HRQOL, and lower depression, anxiety, PD, and PTSD in participants living with a cancer diagnosis. Future investigations, especially cohort studies and RCTs with rigorous study designs, consistent measurements of psychosocial outcomes, and comparable overall healthy lifestyle scoring systems are needed to provide more robust evidence.

## Supplementary Information

Below is the link to the electronic supplementary material.

Supplementary file1 (DOCX 286 KB)

## Data Availability

The datasets were derived from public sources and all data are incorporated into the article and its online supplementary material. The software code used in this study is available from the first author.

## References

[1] Allemani C, et al. Global surveillance of trends in cancer survival 2000–14 (CONCORD-3): analysis of individual records for 37 513 025 patients diagnosed with one of 18 cancers from 322 population-based registries in 71 countries. Lancet. 2018;391(10125):1023–75. 10.1016/s0140-6736(17)33326-3. 29395269 10.1016/S0140-6736(17)33326-3 PMC5879496

[2] Bray F, et al. Global cancer statistics 2022: GLOBOCAN estimates of incidence and mortality worldwide for 36 cancers in 185 countries. CA Cancer J Clin. 2024;74(3):229–63. 10.3322/caac.21834. 38572751 10.3322/caac.21834

[3] Tonorezos E, et al. Prevalence of cancer survivors in the United States. JNCI: J Natl Cancer Inst. 2024;djae135. Advance online publication. 10.1093/jnci/djae135. 10.1093/jnci/djae135 PMC11542986 39002121

[4] De Angelis R, et al. Complete cancer prevalence in Europe in 2020 by disease duration and country (EUROCARE-6): a population-based study. Lancet Oncol. 2024;25(3):293–307. 10.1016/s1470-2045(23)00646-0. 38307102 10.1016/S1470-2045(23)00646-0

[5] Hu S, et al. Mental health outcomes in a population-based cohort of patients with prostate cancer. J Natl Cancer Inst. 2024;116(3):445–54. 10.1093/jnci/djad175. 37867158 10.1093/jnci/djad175 PMC10919332

[6] Carreira H, et al. Associations between breast cancer survivorship and adverse mental health outcomes: a systematic review. J Natl Cancer Inst. 2018;110(12):1311–27. 10.1093/jnci/djy177. 30403799 10.1093/jnci/djy177 PMC6292797

[7] Lemij AA, et al. Mental health outcomes in older breast cancer survivors: five-year follow-up from the CLIMB study. Eur J Cancer. 2023;187:87–95. 10.1016/j.ejca.2023.04.001. 37130464 10.1016/j.ejca.2023.04.001

[8] Hagatulah N, et al. Perinatal depression and risk of mortality: nationwide, register based study in Sweden. BMJ. 2024;384:e075462. 10.1136/bmj-2023-075462. 38199643 10.1136/bmj-2023-075462 PMC10777893

[9] Révész D, et al. Associations between alcohol consumption and anxiety, depression, and health-related quality of life in colorectal cancer survivors. J Cancer Surviv. 2022;16(5):988–97. 10.1007/s11764-021-01090-y. 34529261 10.1007/s11764-021-01090-y PMC9489554

[10] Yan G, et al. Trends in the prevalence and treatment of comorbid depression among US adults with and without cancer, 2005–2020. J Affect Disord. 2023;340:743–50. 10.1016/j.jad.2023.08.091. 37598717 10.1016/j.jad.2023.08.091

[11] Rethorst CD, et al. Considering depression as a secondary outcome in the optimization of physical activity interventions for breast cancer survivors in the PACES trial: a factorial randomized controlled trial. Int J Behav Nutr Phys Act. 2023;20(1):47. 10.1186/s12966-023-01437-x. 37081460 10.1186/s12966-023-01437-x PMC10120257

[12] Chen X, et al. Exercise, tea consumption, and depression among breast cancer survivors. J Clin Oncol. 2010;28(6):991–8. 10.1200/jco.2009.23.0565. 20048185 10.1200/JCO.2009.23.0565 PMC2834436

[13] Smits A, et al. Body mass index and the quality of life of endometrial cancer survivors–a systematic review and meta-analysis. Gynecol Oncol. 2015;137(1):180–7. 10.1016/j.ygyno.2015.01.540. 25636459 10.1016/j.ygyno.2015.01.540

[14] Webster RT, et al. Health behavior profiles in young survivors of childhood cancer: findings from the St Jude Lifetime Cohort Study. Cancer. 2023;129(13):2075–83. 10.1002/cncr.34749. 36943740 10.1002/cncr.34749 PMC10258145

[15] Iyer NS, et al. Health behaviors in cervical cancer survivors and associations with quality of life. Clin Ther. 2016;38(3):467–75. 10.1016/j.clinthera.2016.02.006. 26926320 10.1016/j.clinthera.2016.02.006 PMC4799758

[16] Liberati A, et al. The PRISMA statement for reporting systematic reviews and meta-analyses of studies that evaluate health care interventions: explanation and elaboration. PLoS Med. 2009;6(7):e1000100. 10.1371/journal.pmed.1000100. 19621070 10.1371/journal.pmed.1000100 PMC2707010

[17] NIH. National heart lung and blood institute, Study Quality Assessment Tools. 2018 [cited 2024 22,5]; Available from: https://www.nhlbi.nih.gov/health-topics/study-quality-assessment-tools.

[18] Higgins JP, et al. The Cochrane Collaboration’s tool for assessing risk of bias in randomised trials. BMJ. 2011;343:d5928. 10.1136/bmj.d5928. 22008217 10.1136/bmj.d5928 PMC3196245

[19] Makovski TT, et al. Multimorbidity and quality of life: systematic literature review and meta-analysis. Ageing Res Rev. 2019;53:100903. 10.1016/j.arr.2019.04.005. 31048032 10.1016/j.arr.2019.04.005

[20] Sharma S, et al. Diet quality and risk of SARS-CoV-2 infection or COVID-19: a systematic review of observational studies. Adv Nutr. 2023;14(6):1596–616. 10.1016/j.advnut.2023.09.006. 37748553 10.1016/j.advnut.2023.09.006 PMC10721534

[21] Zhang YB, et al. Combined lifestyle factors, incident cancer, and cancer mortality: a systematic review and meta-analysis of prospective cohort studies. Br J Cancer. 2020;122(7):1085–93. 10.1038/s41416-020-0741-x. 32037402 10.1038/s41416-020-0741-x PMC7109112

[22] Song R, et al. Different operationalizations of the 2018 WCRF/AICR cancer prevention recommendations and risk of cancer. Br J Cancer. 2023;129(6):982–92. 10.1038/s41416-023-02314-x. 37500788 10.1038/s41416-023-02314-x PMC10491614

[23] Van Blarigan EL, et al. Association of survival with adherence to the American Cancer Society nutrition and physical activity guidelines for cancer survivors after colon cancer diagnosis: the CALGB 89803/alliance trial. JAMA Oncol. 2018;4(6):783–90. 10.1001/jamaoncol.2018.0126. 29710284 10.1001/jamaoncol.2018.0126 PMC6145685

[24] Ware JE Jr. SF-36 health survey update. Spine (Phila Pa 1976). 2000;25(24):3130–9. 10.1097/00007632-200012150-00008. 11124729 10.1097/00007632-200012150-00008

[25] Rosenthal R, Rubin DB. Meta-analytic procedures for combining studies with multiple effect sizes. Psychol Bull. 1986;99(3):400–6. 10.1037/0033-2909.99.3.400.

[26] Chia EM, et al. Utility and validity of the self-administered SF-36: findings from an older population. Ann Acad Med Singap. 2006;35(7):461–7. 16902721

[27] Zeraatkar D, et al. Red and processed meat consumption and risk for all-cause mortality and cardiometabolic outcomes: a systematic review and meta-analysis of cohort studies. Ann Intern Med. 2019;171(10):703–10. 10.7326/m19-0655. 31569213 10.7326/M19-0655

[28] Olson JL, et al. Lifestyle behaviors and health-related quality of life in cancer survivors: a latent class analysis. Health Educ Behav. 2024;51(3):341–51. 10.1177/10901981231203978. 37830356 10.1177/10901981231203978 PMC11092290

[29] Glasgow TE, McGuire KP, Fuemmeler BF. Eat, sleep, play: health behaviors and their association with psychological health among cancer survivors in a nationally representative sample. BMC Cancer. 2022;22(1):648. 10.1186/s12885-022-09718-7. 35698055 10.1186/s12885-022-09718-7 PMC9190125

[30] Chung J, et al. Modifiable lifestyle behaviours impact the health-related quality of life of bladder cancer survivors. BJU Int. 2020;125(6):836–42. 10.1111/bju.15007. 31977152 10.1111/bju.15007 PMC7496791

[31] van Veen MR, et al. Adherence to the World Cancer Research Fund/American Institute for Cancer Research recommendations for cancer prevention is associated with better health-related quality of life among long-term colorectal cancer survivors: results of the PROFILES registry. Support Care Cancer. 2019;27(12):4565–74. 10.1007/s00520-019-04735-y. 30927111 10.1007/s00520-019-04735-y PMC6825038

[32] Zhang FF, et al. Lifestyle factors and health-related quality of life in adult survivors of childhood cancer: a report from the St Jude Lifetime Cohort Study. Cancer. 2018;124(19):3918–23. 10.1002/cncr.31647. 30204245 10.1002/cncr.31647 PMC6226352

[33] Breedveld-Peters JJL, et al. Colorectal cancers survivors’ adherence to lifestyle recommendations and cross-sectional associations with health-related quality of life. Br J Nutr. 2018;120(2):188–97. 10.1017/s0007114518000661. 29658446 10.1017/S0007114518000661

[34] Spector DJ, et al. Are lifestyle behavioral factors associated with health-related quality of life in long-term survivors of non-Hodgkin lymphoma? Cancer. 2015;121(18):3343–51. 10.1002/cncr.29490. 26036473 10.1002/cncr.29490 PMC4560969

[35] Song S, et al. Adherence to guidelines for cancer survivors and health-related quality of life among Korean breast cancer survivors. Nutrients. 2015;7(12):10307–19. 10.3390/nu7125532. 26690215 10.3390/nu7125532 PMC4690084

[36] Schlesinger S, et al. Lifestyle factors and health-related quality of life in colorectal cancer survivors. Cancer Causes Control. 2014;25(1):99–110. 10.1007/s10552-013-0313-y. 24158780 10.1007/s10552-013-0313-y

[37] Inoue-Choi M, et al. Adherence to the World Cancer Research Fund/American Institute for Cancer Research recommendations for cancer prevention is associated with better health-related quality of life among elderly female cancer survivors. J Clin Oncol. 2013;31(14):1758–66. 10.1200/jco.2012.45.4462. 23569318 10.1200/JCO.2012.45.4462 PMC3641697

[38] von Gruenigen VE, et al. Lifestyle challenges in endometrial cancer survivorship. Obstet Gynecol. 2011;117(1):93–100. 10.1097/AOG.0b013e31820205b3. 21173649 10.1097/AOG.0b013e31820205b3

[39] Grimmett C, et al. Lifestyle and quality of life in colorectal cancer survivors. Qual Life Res. 2011;20(8):1237–45. 10.1007/s11136-011-9855-1. 21286822 10.1007/s11136-011-9855-1

[40] Blanchard CM, Courneya KS, Stein K. Cancer survivors’ adherence to lifestyle behavior recommendations and associations with health-related quality of life: results from the American Cancer Society’s SCS-II. J Clin Oncol. 2008;26(13):2198–204. 10.1200/jco.2007.14.6217. 18445845 10.1200/JCO.2007.14.6217

[41] Vidra N, et al. Longitudinal associations of adherence to lifestyle recommendations and health-related quality of life in patients with non-muscle invasive bladder cancer. Int J Cancer. 2023;152(10):2032–42. 10.1002/ijc.34418. 36594579 10.1002/ijc.34418

[42] Kim B, et al. Lower risk of depression after smoking cessation and alcohol abstinence in patients with gastric cancer who underwent gastrectomy: a population-based, nationwide cohort study. Cancer. 2023;129(18):2893–903. 10.1002/cncr.34849. 37195133 10.1002/cncr.34849

[43] Kenkhuis MF, et al. Longitudinal associations of adherence to the World Cancer Research Fund/American Institute for Cancer Research (WCRF/AICR) lifestyle recommendations with quality of life and symptoms in colorectal cancer survivors up to 24 months post-treatment. Cancers (Basel). 2022;14(2):417. 10.3390/cancers14020417. 35053579 10.3390/cancers14020417 PMC8774035

[44] Eyl-Armbruster RE, et al. Change toward healthier lifestyles is associated with better health-related quality of life in long-term colorectal cancer survivors. J Natl Compr Canc Netw. 2022;20(11):1233-1243.e10. 10.6004/jnccn.2022.7049. 36351340 10.6004/jnccn.2022.7049

[45] Lei YY, et al. Adherence to the World Cancer Research Fund/American Institute for Cancer Research Guideline is associated with better health-related quality of life among Chinese patients with breast cancer. J Natl Compr Canc Netw. 2018;16(3):275–85. 10.6004/jnccn.2017.7202. 29523666 10.6004/jnccn.2017.7202

[46] Seib C, et al. Improving health-related quality of life in women with breast, blood, and gynaecological cancer with an eHealth-enabled 12-week lifestyle intervention: the women’s wellness after cancer program randomised controlled trial. BMC Cancer. 2022;22(1):747. 10.1186/s12885-022-09797-6. 35804322 10.1186/s12885-022-09797-6 PMC9264489

[47] Hawkes AL, et al. Effects of a telephone-delivered multiple health behavior change intervention (CanChange) on health and behavioral outcomes in survivors of colorectal cancer: a randomized controlled trial. J Clin Oncol. 2013;31(18):2313–21. 10.1200/jco.2012.45.5873. 23690410 10.1200/JCO.2012.45.5873

[48] Gao R, et al. Exercise intervention for post-treatment colorectal cancer survivors: a systematic review and meta-analysis. J Cancer Surviv. 2020;14(6):878–93. 10.1007/s11764-020-00900-z. 32533468 10.1007/s11764-020-00900-z

[49] Barchitta M, et al. The effects of diet and dietary interventions on the quality of life among breast cancer survivors: a cross-sectional analysis and a systematic review of experimental studies. Cancers (Basel). 2020;12(2):322. 10.3390/cancers12020322. 32019093 10.3390/cancers12020322 PMC7072135

[50] Yeganeh L, et al. The effects of lifestyle and behavioural interventions on cancer recurrence, overall survival and quality of life in breast cancer survivors: a systematic review and network meta-analysis. Maturitas. 2024;185:107977. 10.1016/j.maturitas.2024.107977. 38574414 10.1016/j.maturitas.2024.107977

[51] Lahart IM, et al. Physical activity for women with breast cancer after adjuvant therapy. Cochrane Database Syst Rev. 2018;1(1):Cd11292. 10.1002/14651858.CD011292.pub2. 10.1002/14651858.CD011292.pub2 PMC6491330 29376559

[52] Fong DY, et al. Physical activity for cancer survivors: meta-analysis of randomised controlled trials. BMJ. 2012;344:e70. 10.1136/bmj.e70. 22294757 10.1136/bmj.e70 PMC3269661

[53] Yeganeh L, et al. Effects of lifestyle modification on cancer recurrence, overall survival and quality of life in gynaecological cancer survivors: a systematic review and meta-analysis. Maturitas. 2018;111:82–9. 10.1016/j.maturitas.2018.03.001. 29673836 10.1016/j.maturitas.2018.03.001

[54] Forbes CC, et al. Physical activity and nutrition interventions for older adults with cancer: a systematic review. J Cancer Surviv. 2020;14(5):689–711. 10.1007/s11764-020-00883-x. 32328828 10.1007/s11764-020-00883-x PMC7473955

[55] Dinas PC, et al. Combined effects of physical activity and diet on cancer patients: a systematic review and meta-analysis. Nutrients. 2024;16(11):1749. 10.3390/nu16111749. 38892682 10.3390/nu16111749 PMC11175154

[56] Odynets T, Briskin Y, Todorova V. Effects of different exercise interventions on quality of life in breast cancer patients: a randomized controlled trial. Integr Cancer Ther. 2019;18:1534735419880598. 10.1177/1534735419880598. 31625419 10.1177/1534735419880598 PMC6801883

[57] Wang X, et al. Combined healthy lifestyle and depressive symptoms: a meta-analysis of observational studies. J Affect Disord. 2021;289:144–50. 10.1016/j.jad.2021.04.030. 33979724 10.1016/j.jad.2021.04.030

[58] Miller NE, et al. Psychological distress and health behaviours in people living with and beyond cancer: a cross-sectional study. Sci Rep. 2024;14(1):15367. 10.1038/s41598-024-66269-6. 38965364 10.1038/s41598-024-66269-6 PMC11224398

[59] World Cancer Research Fund/American Institute for Cancer Research. Food, nutrition, physical activity, and the prevention of cancer: a global perspective. American Institute for Cancer Research. 2007;1.

[60] World Cancer Research Fund/American Institute for Cancer Research. Diet, nutrition, physical activity, and cancer: a global perspective. Continuous Update Project Expert Report. 2018 [cited 2024 October]. Available from: dietandcancerreport.org.

