# Supplementary material for: Combined healthy lifestyle factors and psychosocial outcomes among cancer survivors: a systematic review and meta-analysis
Source: J Cancer Surviv. 2024 Nov 9;20(3):1023–34. doi: 10.1007/s11764-024-01705-0 (PMC13144231; doi:10.1007/s11764-024-01705-0)
Supplement: Supplementary file 1 — Supplementary file1 (DOCX 286 KB) [file 11764_2024_1705_MOESM1_ESM.docx]

**Supplementary materials**

**Title: Combined healthy lifestyle factors and psychosocial outcomes among cancer survivors: a systematic review and meta-analysis**

Chunsu Zhu^1,2^, Zhiwei Lian ^1,2^, Volker Arndt^1^, Melissa S. Y. Thong^1*^

**Corresponding author**

Melissa S. Y. Thong, Unit of Cancer Survivorship, German Cancer Research Center (DKFZ), Im Neuenheimer Feld 280, 69120, Heidelberg, Germany. Telephone: +49 6221 42-2334. Email: [m.thong@dkfz-heidelberg.de](mailto:m.thong@dkfz-heidelberg.de)

**Journal of Cancer Survivorship**

**Contents**

Table S1. Search strategies

Table S2. The items of National Institute of Health Quality Assessment Tool for Observational Cohort and Cross-sectional Studies

Table S3. The components of combined healthy lifestyle.

Table S4. The quality assessment of cohort studies

Table S5. The quality assessment of cross-sectional studies

Table S6. The quality assessment of randomized controlled trials

Figure S1. Meta-analysis on cross-sectional studies examining the association between combined healthy lifestyle score and HRQOL.

Table S1. Search strategies

| Search | Query |
| --- | --- |
| #1 | Search ("survivor*" OR "patient*") |
| #2 | Search ("Cancer" OR "Neoplasm" OR "carcinoma") |
| #3 | Search #1 AND#2 |
| #4 | Search ("combination*" OR "combined" OR "composite" OR "integrated" OR "interaction*" OR "joint effect*" OR "score*" OR "adherence to" OR "adhere to" OR "adhered to" OR "collective" OR "cumulative" OR "multiple") |
| #5 | Search ("life style*" OR "risk reduction behavior*" OR "health behavior*" OR "health factor*" OR "lifestyle*" OR "low risk*" OR "prevention guideline*" OR "protective factor*" OR "risk reduction behaviour*" OR "health behaviour*" OR "healthy behavior*" OR "healthy behaviour*" OR "risk behavior*" OR "risk behaviour*" OR "modifiable factor*") |
| #6 | Search #4 AND #5 |
| #7 | Search ("life quality" OR "health related quality of life" OR "health related quality of life" OR "HRQOL" OR "quality of life" OR "HRQOL") |
| #8 | Search ("depressive disorder" OR "affective symptoms" OR "mood disorders" OR "dysthymic disorder" OR "depression" OR "depress*" OR "dysthymia" OR "affective disorder*" OR "MDD") |
| #9 | Search ("Anxiety" OR "social anxiety" OR "anxiety disorder*" OR "anxiety state*" OR "Nervousness" OR "Anxiousness") |
| #10 | Search ("post traumatic" OR "complex trauma*" OR "disaster distress*" OR "disaster stress*" OR "postdisaster distress*" OR "postdisaster stress*" OR "posttraumatic distress*" OR "posttraumatic disorder*" OR "posttraumatic stress*" OR "ptsd*" OR "traumatic distress*" OR "traumatic disorder*" OR "traumatic stress*" OR "trauma and stressor related disorder*" OR "PTSD" OR “PTS”) |
| #11 | Search ("mental health" OR "mental illness" OR "mental disorder*" OR "wellbeing" OR "psychological symptoms" OR "psychological health" OR "psychological functioning" OR "psychosocial outcomes" OR "psychological distress" OR "somatic complaints" OR "somatization") |
| #12 | Search #7 OR #8 OR #9 OR #10 OR #11 |
| #13 | Search #3 AND #6 AND #12 |

Table S2. The items of National Institute of Health Quality Assessment Tool for Observational Cohort and Cross-sectional Studies

| **Items** | **Contents** |
| --- | --- |
| 1 | Was the research question or objective in this paper clearly stated? |
| 2 | Was the study population clearly specified and defined? |
| 3 | Was the participation rate of eligible persons at least 50%? |
| 4 | Were all the subjects selected or recruited from the same or similar populations (including the same time period)? Were inclusion and exclusion criteria for being in the study prespecified and applied uniformly to all participants? |
| 5 | Was a sample size justification, power description, or variance and effect estimates provided? |
| 6 | For the analyses in this paper, were the exposure(s) of interest measured prior to the outcome(s) being measured? |
| 7 | Was the timeframe sufficient so that one could reasonably expect to see an association between exposure and outcome if it existed? |
| 8 | For exposures that can vary in amount or level, did the study examine different levels of the exposure as related to the outcome (e.g., categories of exposure, or exposure measured as continuous variable)? |
| 9 | Were the exposure measures (independent variables) clearly defined, valid, reliable, and implemented consistently across all study participants? |
| 10 | Was the exposure(s) assessed more than once over time? |
| 11 | Were the outcome measures (dependent variables) clearly defined, valid, reliable, and implemented consistently across all study participants? |
| 12 | Were the outcome assessors blinded to the exposure status of participants? |
| 13 | Was loss to follow-up after baseline 20% or less? |
| 14 | Were key potential confounding variables measured and adjusted statistically for their impact on the relationship between exposure(s) and outcome(s)? |

Table S3. The components of combined healthy lifestyle

| Author (year) | Components of HLS | | | | | | | Guidelines for  HLS | HLS |
| --- | --- | --- | --- | --- | --- | --- | --- | --- | --- |
|  | PA | Diet | BW | Alcohol | Smoking | Sleep | SB |  |  |
| Vidra (2023) | × | × | × | × |  |  |  | WCRF/AICR | 0-7 |
| Kim (2023) | × |  |  | × | × |  |  | Basic summing | 0-3 |
| Kenkhuis (2022) | × | × | × |  |  |  | × | WCRF/AICR | 0-7 |
| Eyl-Armbruster (2022) | × | × | × | × | × |  |  | Basic summing | 0-5 |
| Lei (2018) | × | × | × |  |  |  |  | WCRF/AICR | 0-6 |
| Hawkes (2013) | × | × | × | × | × |  |  | NA | MLI vs. UC |
| Seib (2022) | × | × |  | × | × | × |  | NA | MLI vs. UC |
| Olson (2023) | × | × |  |  |  | × |  | Other criteria | Healthiest vs. least healthy |
| Glasgow (2022) | × | × |  |  |  | × | × | Basic summing | 0-7 |
| Zhang (2018) | × | × | × | × | × |  |  | ACS | 0-6 |
| Breedveld-Peters (2018) | × | × | × |  |  |  |  | WCRF/AICR | 0-10 |
| Iyer (2016) | × |  |  | × | × |  |  | Basic summing | 0-3 |
| Spector (2015) | × | × | × |  | × |  |  | ACS | 0-4 |
| Schlesinger (2014) | × | × | × |  | × |  |  | Basic summing | 0-4 |
| Inoue-Choi (2013) | × | × | × | × |  |  |  | WCRF/AICR | 0-7 |
| Gruenigen (2011) | × | × |  |  | × |  |  | Basic summing | 0-3 |
| Grimmett (2011) | × | × |  | × | × |  |  | Basic summing | 0-4 |
| Blanchard (2008) | × | × |  |  | × |  |  | Basic summing | 0-3 |
| Veen (2019) | × | × | × | × |  |  |  | WCRF/AICR | 0-8 |
| Song (2015) | × | × | × |  |  |  |  | ACS, WCRF/AICR | 0–6, 0–12 |
| Chung (2020) | × | × | × | × | × |  |  | Basic summing | 0-5 |

WCRF/AICR: World Cancer Research Fund/American Institute for Cancer Research (WCRF/AICR); ACSNPAG: American Cancer Society’s Nutrition and Physical Activity Guidelines; ACS: American Cancer Society; PA: physical activity; BW: body weight; HLS: healthy lifestyle score; SB: sedentary behavior; MLI: multiple lifestyle intervention; UC: usual care.

Table S4. The quality assessment of cohort studies

| **Author (year)** | **Item**  **1** | **Item**  **2** | **Item**  **3** | **Item**  **4** | **Item**  **5** | **Item**  **6** | **Item**  **7** | **Item**  **8** | **Item**  **9** | **Item**  **10** | **Item**  **11** | **Item**  **12** | **Item**  **13** | **Item**  **14** | **Total** |
| --- | --- | --- | --- | --- | --- | --- | --- | --- | --- | --- | --- | --- | --- | --- | --- |
| Vidra(2023) | 1 | 1 | 1 | 1 | 1 | 0 | 1 | 1 | 1 | 1 | 1 | 0 | 1 | 1 | 12 |
| Kim(2023) | 1 | 1 | 0 | 1 | 1 | 1 | 1 | 0 | 1 | 1 | 1 | 0 | 0 | 0 | 9 |
| Kenkhuis(2022) | 1 | 1 | 1 | 1 | 1 | 0 | 1 | 0 | 1 | 1 | 1 | 0 | 1 | 1 | 11 |
| Eyl-Armbruster(2022) | 1 | 1 | 1 | 1 | 1 | 1 | 1 | 0 | 1 | 1 | 1 | 0 | 1 | 1 | 12 |
| Lei(2018) | 1 | 1 | 1 | 1 | 1 | 0 | 1 | 1 | 1 | 1 | 1 | 0 | 1 | 1 | 12 |

Table S5. The quality assessment of cross-sectional studies

| **Author (year)** | **Item 1** | **Item 2** | **Item 3** | **Item 4** | **Item 5** | **Item 9** | **Item 11** | **Item 14** | **Total** |
| --- | --- | --- | --- | --- | --- | --- | --- | --- | --- |
| Breedveld-Peters(2018) | 1 | 1 | 0 | 1 | 0 | 1 | 1 | 1 | 6 |
| Iyer(2016) | 1 | 1 | 0 | 1 | 1 | 0 | 1 | 1 | 6 |
| Spector(2015) | 1 | 1 | 1 | 1 | 1 | 1 | 1 | 1 | 8 |
| Schlesinger(2014) | 1 | 1 | 1 | 1 | 1 | 1 | 1 | 1 | 8 |
| Inoue-Choi(2013) | 1 | 1 | 1 | 1 | 1 | 1 | 1 | 1 | 8 |
| Gruenigen(2011) | 1 | 1 | 1 | 1 | 0 | 1 | 1 | 0 | 6 |
| Grimmett(2011) | 1 | 1 | 0 | 1 | 0 | 1 | 1 | 1 | 6 |
| Blanchard(2008) | 1 | 1 | 1 | 1 | 1 | 0 | 1 | 1 | 7 |
| Veen(2019) | 1 | 1 | 1 | 1 | 1 | 1 | 1 | 1 | 8 |
| Song(2015) | 1 | 1 | 1 | 1 | 0 | 1 | 1 | 1 | 7 |
| Chung(2020) | 1 | 1 | 1 | 1 | 0 | 1 | 1 | 0 | 6 |
| Webster(2023) | 1 | 1 | 1 | 1 | 1 | 0 | 1 | 1 | 7 |
| Olson(2023) | 1 | 1 | 0 | 1 | 1 | 0 | 1 | 0 | 5 |
| Glasgow(2022) | 1 | 1 | 0 | 1 | 1 | 0 | 1 | 1 | 6 |
| Zhang(2018) | 1 | 1 | 1 | 1 | 1 | 1 | 1 | 1 | 8 |

Table S6. The quality assessment of randomized controlled trials

| **Bias domain** | **Level of bias** | **Support for judgement** |
| --- | --- | --- |
| **Seib (2022)** |  |  |
| Random sequence generation | Low risk | Quote: “After baseline assessment, three hundred and fifty-one women previously treated for breast, blood or gynaecological cancer were randomly assigned to either an intervention or usual care arm using permuted block randomisation.” |
| Allocation concealment |  |  |
| Blinding of participants and researchers | High risk | Quote:" While blinding of participants was not possible.." |
| Blinding of outcome assessment | Low risk | Quote:" While blinding of participants was not possible, the trial statistician  and study staff (except for the trial coordinator and those who delivered the intervention) were unaware of group allocation." |
| Incomplete outcome data | Unclear risk | Quote:" A computer-generated allocation sequence using blocks of varying length was developed by the trial statistician, and randomization was performed by the trial coordinator, who logged into a secure server to obtain the next allocation." |
| Selective reporting | Low risk | All prespecified outcomes were reported. |
| Other bias | Low risk | Losses to follow-up were presented clearly and per-protocol (PP) and intent-to-treat (ITT) were performed. |
| **Hawkes (2013)** |  |  |
| Random sequence generation | Low risk | Quote:" participants were randomly assigned at a ratio of one to one using a computer-generated random number sequence." |
| Allocation concealment | Low risk | Quote:" The allocation sequence was generated by CanChange computer application developers and concealed from project investigators and from the project manager who assigned participants to groups." |
| Blinding of participants and researchers | Low risk | Quote:" Data were collected by computer-assisted telephone interview by dedicated staff who were blinded to group assignment." |
| Blinding of outcome assessment | Low risk | Data were collected by computer-assisted telephone interview by dedicated staff who were blinded to group assignment |
| Incomplete outcome data | Low risk | Losses to follow-up were presented clearly. |
| Selective reporting | Low risk | All prespecified outcomes were reported. |
| Other bias | Unclear risk | Quote:" Comparison of the treatment groups on baseline characteristics revealed that the HC group was significantly younger than the UC group." |


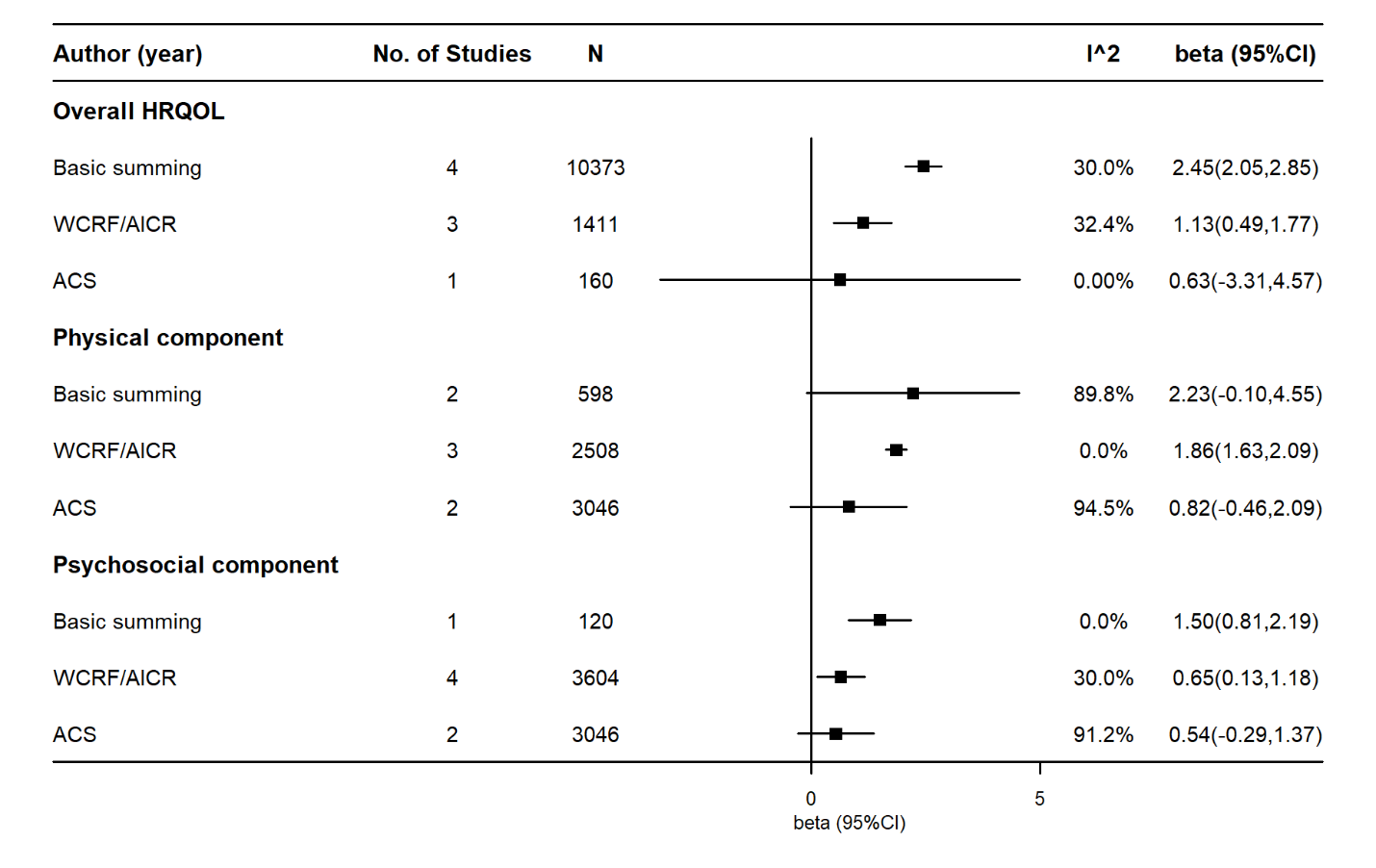


Figure S1. Meta-analysis on cross-sectional studies examining the association between combined healthy lifestyle score and HRQOL. HRQOL: health-related quality of life; CI: confidence interval. Beta coefficient, depicting the average increase in outcome scores with per-unit increase in the combined healthy lifestyle score.
